# Preparing medical first responders for crises: a systematic literature review of disaster training programs and their effectiveness

**DOI:** 10.1186/s13049-022-01056-8

**Published:** 2022-12-24

**Authors:** Anke S. Baetzner, Rafael Wespi, Yannick Hill, Lina Gyllencreutz, Thomas C. Sauter, Britt-Inger Saveman, Stefan Mohr, Georg Regal, Cornelia Wrzus, Marie O. Frenkel

**Affiliations:** 1 Institute of Sports and Sports Sciences, Heidelberg University, Heidelberg, Germany; 2 Department of Emergency Medicine, Inselspital, University Hospital, University of Bern, Bern, Switzerland; 3 Department of Human Movement Sciences, Faculty of Behavioral and Movement Sciences, Vrije Universiteit Amsterdam, Amsterdam Movement Sciences, Amsterdam, Netherlands; 4 Department of Nursing, Umeå University, Umeå, Sweden; 5 Department of Surgical and Perioperative Sciences, Umeå University, Umeå, Sweden; 6 Department of Anesthesiology, University Hospital Heidelberg, Heidelberg, Germany; 7 Center for Technology Experience, AIT Austrian Institute of Technology, Vienna, Austria; 8 Psychological Institute and Network Aging Research, Heidelberg University, Heidelberg, Germany; 9 Institute of Brain and Behaviour Amsterdam, Amsterdam, Netherlands; 10 Lyda Hill Institute for Human Resilience, Colorado Springs, USA; 11 Graduate School for Health Sciences, University of Bern, Bern, Switzerland

**Keywords:** Emergency medical technicians, Emergency medicine, Mass casualty incident, Medical education, Mixed reality, Paramedics, Performance, Prehospital care, Simulation, Virtual reality

## Abstract

**Background:**

Adequate training and preparation of medical first responders (MFRs) are essential for an optimal performance in highly demanding situations like disasters (e.g., mass accidents, natural catastrophes). The training needs to be as effective as possible, because precise and effective behavior of MFRs under stress is central for ensuring patients’ survival and recovery. This systematic review offers an overview of scientifically evaluated training methods used to prepare MFRs for disasters. It identifies different effectiveness indicators and provides an additional analysis of how and to what extent the innovative training technologies virtual (VR) and mixed reality (MR) are included in disaster training research.

**Methods:**

The systematic review was conducted according to the PRISMA guidelines and focused specifically on (quasi-)experimental studies published between January 2010 and September 2021. The literature search was conducted via Web of Science and PubMed and led to the inclusion of 55 articles.

**Results:**

The search identified several types of training, including traditional (e.g., lectures, real-life scenario training) and technology-based training (e.g., computer-based learning, educational videos). Most trainings consisted of more than one method. The effectiveness of the trainings was mainly assessed through pre-post comparisons of knowledge tests or self-reported measures although some studies also used behavioral performance measures (e.g., triage accuracy). While all methods demonstrated effectiveness, the literature indicates that technology-based methods often lead to similar or greater training outcomes than traditional trainings. Currently, few studies systematically evaluated immersive VR and MR training.

**Conclusion:**

To determine the success of a training, proper and scientifically sound evaluation is necessary. Of the effectiveness indicators found, performance assessments in simulated scenarios are closest to the target behavior during real disasters. For valid yet inexpensive evaluations, objectively assessible performance measures, such as accuracy, time, and order of actions could be used. However, performance assessments have not been applied often. Furthermore, we found that technology-based training methods represent a promising approach to train many MFRs repeatedly and efficiently. These technologies offer great potential to supplement or partially replace traditional training. Further research is needed on those methods that have been underrepresented, especially serious gaming, immersive VR, and MR.

**Supplementary Information:**

The online version contains supplementary material available at 10.1186/s13049-022-01056-8.

## Background

Natural and man-made disasters such as floods, mass-accidents, and terrorist attacks are ubiquitous and cause loss of life, human suffering, and infrastructural damage [1, 2]. They create particularly demanding situations for emergency services, as they are unforeseen and usually sudden events that exceed local capacity and resources to rescue and care [1]. During such disasters, medical first responders (MFRs), who are responsible for the initial prehospital care in medical emergencies, play a key role [3, 4]. However, numerous healthcare professionals, including MFRs, perceive their preparedness for the response to disasters as inadequate [5]. As previous research indicates, a higher training frequency and better training quality are associated with increased disaster preparedness [5]. To enhance the overall quality of MFR training, the aim of this review is to provide an overview of scientifically evaluated training methods and to examine whether certain methods seem to be particularly effective. Furthermore, indicators used to evaluate the training effectiveness will be identified so that future research can be guided by existing training evaluation methods. Finally, the emergence of new, immersive technologies, including virtual (VR) and mixed reality [MR; 6], has led to the development of new training programs which are becoming increasingly accessible to educators in the medical sector [7]. Therefore, we will draw particular attention to the role of immersive technologies by providing an additional analysis of how and to what extent VR and MR specifically are included in current disaster training research.

MFRs typically include paramedics and emergency medical technicians [3], but the term may also refer to physicians, ambulance specialist nurses, and trained volunteers depending on a country’s emergency medical service systems [8, 9]. During disasters, MFRs take on a variety of tasks such as the initial scene evaluation, triage, medical care, and the transport of patients [3]. They have to perform those tasks under stressful and challenging conditions, such as difficult access to the disaster site, multiple injured people and disruption in communication systems [10]. In order for MFRs to adapt to these unusual conditions, they require specifically tailored training.

Effective training involves a systematic and goal-oriented execution of exercises for the acquisition or increase of specific competences and skills [11]. The general idea of training is to challenge the current level of performance (e.g., higher intensity, higher difficulty, new content) without being too overwhelming, so that the trainee can adapt and reach a higher performance level [11–15]. However, training resources, including time, budget, and facilities, are usually limited. Therefore, training methods must be not only effective, but also match the resources of the rescue organization.

Despite the necessity of adequately preparing MFRs for disasters, no systematic and up-to-date overview of scientifically evaluated training methods and their effectiveness exists. Ingrassia and colleagues conducted an internet-based search via Google and Bing and identified several disaster management curricula at a postgraduate level with a large variety of methods, e.g., lectures and discussion-based exercises [16]. The trainings’ effectiveness, however, was not evaluated. Assessing studies published between 2000 and 2005, Williams and colleagues [17] concluded that the available evidence had not been sufficient to determine whether disaster training can effectively increase the knowledge and skills of MFRs and in-hospital staff. Because these findings are derived from studies conducted more than 15 years ago, new insights have most likely emerged and new training methods may have been added following recent technological advances.

Two of those new methods are VR and MR. In VR training, users are placed inside a simulated, artificial, three-dimensional environment in which they can interact with their digital surroundings [6]. VR can either be screen-based using computer monitors or experienced in more immersive forms: Through head-mounted displays or certain rooms equipped with several large screens or projections on several walls (i.e., CAVE system; 6,18). In contrast, MR combines the real and virtual worlds and refers to the whole spectrum between reality and VR. MR, for example, includes augmented reality (AR) in which users see their real surroundings supplemented with virtual objects [6]. A specific application from the medical field may be the visual insertion of patient information during practice. Given the rapid development of immersive technology, this review provides an additional analysis of the role of VR and MR training.

Altogether, the following research questions are addressed:

1. Which current disaster training methods for MFRs have already been scientifically evaluated?
2. Which effectiveness indicators are used to evaluate MFR disaster training methods?
3. Based on the findings of the reviewed studies, which methods for MFR disaster training seem to be effective?
4. How and to what extent are VR and MR used to prepare MFRs for disasters?

## Methods

The preregistered (osf.io/yn5v3) systematic literature search was conducted in accordance with the PRISMA guidelines [19].

### Search strategy

The search strategy was prepared with support of a medical information specialist to ensure the appropriateness of the search terms. Using the search engines Web of Science and PubMed, we applied search terms such as *health personnel*, *training*, and *disaster* (see Additional file 1 for the search string). To ensure that the results reflect current training methods, the electronic search was limited to studies published between January 2010 and September 2021. A filter limited our results to studies with a full text in English.

### Inclusion and exclusion criteria

We included articles that described a training or training session (e.g., drill, lectures, mixed methods training, etc.) conducted to improve the participants’ *prehospital* disaster response. The training had to address prehospital content, but was allowed to also contain in-hospital topics. Participants had to be MFRs, regardless of whether they were still in training or already had work experience. In addition, to ensure adequate assessments of the effectiveness, we only considered (quasi-)experimental designs in which outcomes were compared to a control or comparison group [i.e., randomized controlled trials (RCTs), non-RCTs and at minimum pre-post testing of the same group; [20, 21]].

We excluded studies that a) did not test the effectiveness of a disaster training for MFRs, b) contained other occupational groups or not sufficiently specified groups (e.g., “others”) in addition to MFRs without reporting separate analyses for the MFRs, c) were not primary studies published in peer-reviewed journals, and d) had no full-text available.

### Selection process

**Fig. 1:**
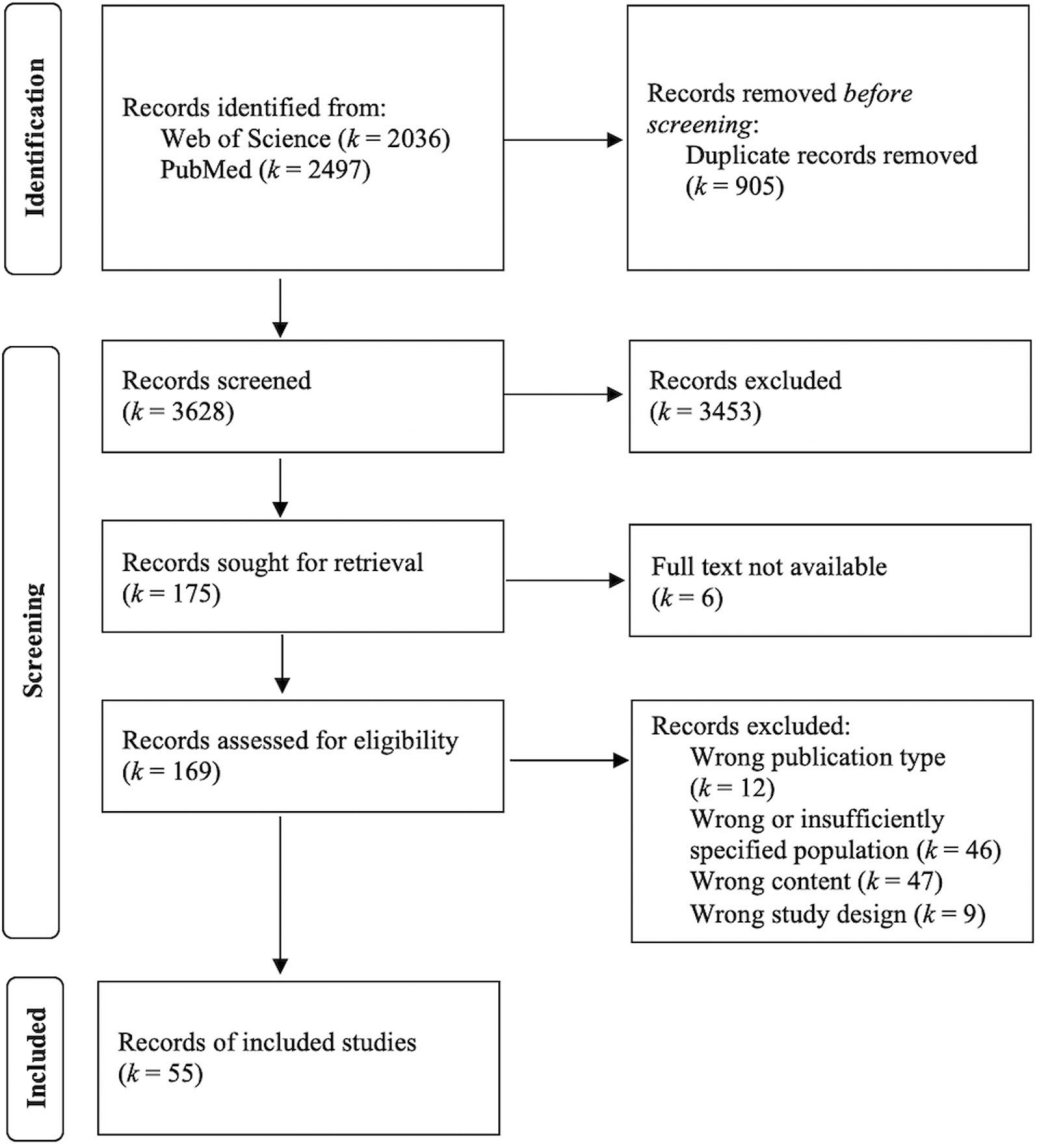
Flow diagram of study selection

The search was conducted on 28th October 2021 and led to 4533 hits (Fig. 1). Duplicates were identified with the software Endnote™ (Version 20.1) and additional visual screening. Two raters (ASB and RW) independently screened the remaining hits and performed the study selection using the web application Rayyan [22]. Discrepancies in the study selection process were resolved by consensus or, if necessary, together with a third rater (YH). Fifty-five studies were included in the review.

### Data collection and analysis

Two raters (ASB and RW) extracted the relevant information for each article. Again, discrepancies were resolved by consensus, and when necessary together with the third rater (YH). Whenever studies used multiple methods at different time points we only considered those applied between the pre- and post-measurement. For trainings evaluated in (non-)RCTs without a pre-test, methods must have been applied before the post-test comparison with control groups. Similarly, only effectiveness indicators with sufficient informative value about training success or failure were considered (i.e., indicators used for pre-post comparisons or for comparisons with control groups). To assess the studies’ quality and risk of bias, we used the Joanna Briggs Institute (JBI) critical appraisal checklists for RCTs and quasi‐experimental studies [23]. The JBI tool for quasi-experimental studies contains nine questions and the JBI tool for experimental studies consists of 13 questions (e.g., “Were outcomes measured in a reliable way?”). There are four possible answer options: *yes*, *no*, *unclear*, *not applicable*. The answer *yes* indicates quality while *no* indicates a risk of bias.

## Results

The majority of studies used a single group pre-post design (*k* = 35). Other study designs included non-RCTs (*k* = 6) and RCTs (*k* = 14) with 15 out of 20 containing pre-post testing (see Table 1 for a full overview and Additional file 1: Table 1 for further information). The sample sizes varied largely between studies (range 6–524). Trainings took place on several continents with the majority of trainings conducted in North America (*k* = 24), followed by Asia (*k* = 18), Europe (*k* = 8), Australia and Africa (both *k* = 2), unclear (*k* = 1). The majority of tested trainings addressed general disaster management or several disaster-related topics (*k* = 31), followed by triage (*k* = 14), trauma management/sonography (*k* = 3) etc. Furthermore, the time spans varied between one day or less (*k* = 22) to up to eight months (*k* = 33).

**Table 1:** Overview of included studies

First author, year	Study design	N^1^	Professions	Training content	Training methods	Duration, timespan	Effectiveness indicators	Effectiveness confirmation
Aghababaeian, 2013 [58]	Non-RCT (pre-post)	144	Paramedics, EMTs	Triage	IG: Educational video CG: Real-life scenario	0.5 h, 1 day	Basic knowledge test	No sign. difference between groups
							Applied knowledge test (triage accuracy)	IG partially better than CG
Alenyo, 2018 [77]	Single group, pre-post	129	Emergency medical service providers	General disaster management	Not specified	NA, NA	Applied knowledge test (triage accuracy)	Yes
Alim, 2015 [78]	Single group, pre-post	309	Nursing students	General disaster management	Not sufficiently clear (“in-class training”)	8 h, 1 day	Basic knowledge test	Yes
Aluisio, 2016 [79]	Single group, pre-post	12	Physicians, nurses	Lower extremity regional anesthesia for earthquake victims	Discussion-based training, practical skills	5.25 h, 1 day	Basic knowledge test	Yes
Andreatta, 2010 [25]	Stratified RCT (pre-post)	15	Physicians	Triage	IG: Lecture, VR with CAVE CG: Lecture, real-life scenario	NA, 1 day	Basic knowledge test	CG better than IG (effect sizes only)
							Observed performance with a self-developed instrument to compose an overall performance score during real-life / VR simulation (e.g., ensure safety on scene, call for additional help, accuracy)	IG better than CG (effect sizes only)
Andreatta, 2015 [34]	Non-RCT (pre-post)	204	Nurses, paramedics, medical students	Cholinergic crisis management	IG (use of virtual animal model): Discussion-based training, real-life scenario, computer-based learning CG (use of virtual human model): Discussion-based training, real-life scenario, computer-based learning	4 h, 1 day	Basic knowledge test	For all indicators: Yes, for both groups (no sign. difference between groups)
							Observed performance with a previously validated instrument [80] to compose an overall performance score during real-life simulation (multiple performance dimensions associated with managing a nerve agent casualty)	
							Self-reports of self-efficacy and state affect	
Bajow, 2016 [63]	Single group, pre-post	29	Medical students	General disaster management	Lecture, discussion-based training, real-life scenario, VR on screen, educational video, debriefing, field visit	53 h, 2 weeks	Basic knowledge test	Yes
Betka, 2021 [49]	Single group, pre-post	17	Medical students, nursing students^2^	General disaster management	Discussion-based training, computer-based learning	NA, NA	Self-rep. interprofessional collaborative competencies	Partially confirmed
							Self-rep. disaster management competence	Yes
							Self-rep. self-confidence in managing disasters	Partially confirmed
Chan, 2010 [71]	Single group, pre-post	138	Nursing students	General disaster management	Lecture, discussion-based training, practical skills, field visit	NA, 2 weeks	Self-rep. competence in disaster nursing	Yes
Chandra, 2014 [52]	Single group, pre-post	76	Professional volunteers	Psychological first aid	Lecture, discussion-based training, educational video	2 h 1 day	Basic knowledge test	No
							Self-rep. capability in using psychological first aid	Yes
Chou, 2021 [53]	Single group, pre-post	48	Medical students	General disaster management	Lecture, discussion-based training, real-life scenario	NA, 2 days	Basic knowledge test	Partially confirmed
							Self-rep. willingness to pursue further training	No
							Interest in disaster training	No
Cicero, 2012 [26]	Single group, pre-post	50	Physicians	Triage	Lecture, real-life scenario, debriefing	3.5 h, 5 months	Observed triage performance during real-life simulation (accuracy)	Yes
Cicero, 2017 [27]	Single group, pre-post	261	Paramedics, EMTs, paramedic students	Triage	Real-life scenario, debriefing, computer-based learning	5 h, 6.5 months	Observed triage performance during real-life simulation (accuracy)	Yes
Cowling, 2021 [45]	Single group, pre-post	26	EMTs in training	General disaster management	Real-life scenario, debriefing	NA, 1 day	Basic knowledge test	No
							Self-rep. knowledge	Yes
							Self-rep. confidence in managing a structural collapse scenario	Yes
Cuttance, 2017 [57]	RCT (only post)	292	Paramedics	Triage	IG1: lecture, other (aid memoire) IG2: lecture IG3: other (aid memoire) CG: no intervention	NA, NA	Applied knowledge test (triage accuracy)	Yes, all IGs improved with the greatest improvements in IG1 and IG3
Dittmar, 2018 [28]	Single group, pre-post	19	Paramedics^2^	Triage	Lecture	0.75 h, 1 day	Observed triage performance during real-life simulation (time + accuracy + overall performance score assessed with a self-developed instrument that incorporated e.g., accuracy, time, airway handling and bleeding control measures)	Yes
Edinger, 2019 [81]	Single group, pre-post	19	Emergency medical service providers	Interacting with individuals with developmental disabilities during disaster response	Computer-based learning	1 h, 1 day	Basic knowledge test	Partially confirmed
							Self-rep. self-efficacy for caring for developmentally disabled individuals	Yes
Farra, 2013 [61]	RCT (pre-post)	47	Nursing students	General disaster management	IG: computer-based learning, VR on screen CG: computer-based learning	NA, NA	Basic knowledge test	IG partially better but CG improved as well (within-group effect of CG not tested for significance)
Fernandez-Pacheco, 2017 [60]	Single group, pre-post	35	Nursing students	Triage	Debriefing with video	NA, NA	Change in self-perception (number of behaviors, moments, thoughts, feelings, strengths + weaknesses being described)	Partially confirmed
Foronda, 2016 [62]	Single group, pre-post	6	Nursing students	Triage	Lecture, VR on screen, debriefing	1.25, 1 day	Applied knowledge test (triage accuracy)	No
Furseth, 2016 [50]	Non-RCT (pre-post)	189	Nursing students, paramedic students	General disaster management	IG with a training focus on handoff communication: lecture, real-life scenario, debriefing CG: lecture, real-life scenario, debriefing	NA, 1 day	Self-rep. attitude towards Interprofessional education and interprofessional healthcare teams	For all indicators: IG partially better
							Self-rep. confidence	
							Satisfaction with the training	
Greco, 2019 [47]	Single group, pre-post	90	Nursing students	Triage	Real-life scenario, debriefing	NA, NA	Self-rep. ethical reasoning confidence	Yes
							perceived importance of ethical reasoning	
Huh, 2019 [41]	RCT (pre-post)	60	Nursing students	General disaster management	IG: Lecture, discussion-based training, practical skills, educational video CG: No intervention	8 h, 4 weeks	Basic knowledge test	For both indicators: Yes
							Self-rep. disaster readiness	
Hutchinson, 2011 [82]	Single group, pre-post	81	Nursing students	General disaster management	Lecture, discussion-based training, real-life scenario, educational video, computer-based learning	NA, NA	Basic knowledge test	Partially confirmed
Ingrassia, 2015 [29]	RCT (pre-post) cross-over design	56	Medical students	Triage	IG: Lecture, VR on screen CG: Lecture, real-life scenario training	NA, 3 days	Triage performance observed during real-life/virtual simulation (accuracy of triage decisions and of decisions about the need for lifesaving treatments + time)	No sign. difference between groups but both groups improved significantly
Ingrassia, 2014 [83]	Single group, pre-post	524	Medical students	General disaster management	Discussion-based training, computer-based learning, debriefing	47 h, 1 month	Basic knowledge test	For both indicators: Yes
							Applied knowledge test (triage accuracy)	
James, 2021 [84]	Single group, pre-post	34	Nursing students	General disaster management	Real-life scenario, debriefing	4 h, 1 day	Self-rep. attitudes towards teamwork in training	Yes
Jones, 2014 [43]	Single group, pre-post	224	Paramedics, EMTs	Active shooter incident response	Lecture, real-life scenario	4 h, 1 day	Self-rep. preparedness and attitudes towards active shooter incident response	Yes (but only descriptive statistics)
Kim, 2020 [55]	Single group, pre-post	34	Nursing students	General disaster management	Lecture, discussion-based training, real-life scenario, debriefing	3 h, 1 day	Self-rep. attitudes towards responding to MCIs	Yes
Knight, 2010 [30]	Non-RCT (only post)	91	Physicians, nurses, paramedics	Triage	IG: lecture, serious gaming with VR on screen CG: lecture, discussion-based training	NA, NA	Observed triage performance during real-life simulation (triage accuracy + compliance with the correct procedure + time)	Accuracy: IG better than CG, step accuracy: IG partially better, time: no sign. difference between groups
Koca, 2020 [42]	RCT (pre-post)	235	Nursing students	General disaster management	IG: computer-based learning CG: no intervention	11 h, 2 weeks	Self-reports of preparedness and self-efficacy	Yes
Koutitas, 2021 [38]	RCT (only post)	30	EMTs	Familiarization with the ambulance bus (AMBUS)	IG1: lecture, VR with HMD IG2: lecture, AR with a head-mounted display CG: lecture, field visit	NA, 1 week	Observed performance in a real ambulance bus (orientation in ambulance bus; accuracy + time + overall performance score calculated from accuracy and time)	Yes, for both IGs with better results in IG1 (only descriptive statistics reported)
Kuhls, 2017 [48]	Single group, pre-post	78	Physicians, nurses^2^	General disaster management	Not specified	8 h, 1 day	Self-rep. confidence to manage disaster scenarios	Yes
Lampi, 2013 [85]	Single group, pre-post	153	Physicians	Trauma life support	Lecture, discussion-based training, practical skills	NA, NA	Applied knowledge test (triage accuracy)	No
Lennquist Montán, 2015 [86]	Single group, pre-post	83	Emergency medical services providers^2^	General disaster management	Discussion-based training	NA, 3 days	Self-rep. knowledge	For both indicators: Yes
							Self-rep. skills	
Ma, 2021 [24]	RCT (pre-post)	104	Nursing students	General disaster management	IG: screen-based serious gaming CG: discussion-based training, real-life scenario, practical skills, debriefing	2 h, NA	Self-rep. competence in disaster nursing	IG better than CG
Merlin, 2010 [54]	Single group, pre-post	46	Medical students	General disaster management	Lecture, practical skills, field visit/clerkship	34.5 h, 4 weeks	Self-rep. knowledge	For both indicators: Yes
							Self-rep. opinions about prehospital issues	
Mills, 2020 [31]	RCT (only post) cross-over design	29	Paramedic students	Triage	IG: VR with HMD CG: real-life scenario	NA, 1 day	Triage performance (accuracy) during real-life/VR simulation	No sign. difference between groups
							Immersion (via heart-rate and self-rep.)	Greater immersion in real-life training
							Learning satisfaction	No sign. difference between groups
Motola, 2015 [35]	RCT (pre-post)	91	Paramedics	Managing CBRNE incidents	IG: Educational video Waiting CG: no intervention	NA, NA	Basic knowledge test	Yes
							Observed performance in treating a CBRNE patient (overall performance score assessed with an instrument based on a previous study; [87])	Partially confirmed
Paddock, 2015 [37]	Stratified RCT (pre-post)	36	Physicians, nurses, paramedics, EMTs	Prehospital focused assessment with sonography in trauma	IG1: Educational video, computer-based learning CG: Educational video, practical skills IG2: Both of the trainings above	4 h, 1 day	Basic knowledge test	No sign. difference between groups but all trainings led to significant improvement
							Observed sonography performance (overall performance scores for image acquisition and interpretation assessed with a self-developed instruments)	no sign. difference between groups
Phattaharapornjaroen, 2020 [88]	Single group, pre-post	52	Physicians	General disaster management	Discussion-based training	NA, 2 days	Self-rep. knowledge	Yes
Pollard, 2015 [89]	Single group, pre-post	41	Medical students	General disaster management	Lecture, real-life scenario, computer-based learning	NA, 8 months	Basic knowledge test	Yes
Pouraghaei, 2017 [36]	Single group, pre-post	205	EMTs	Triage	Lecture, discussion-based training	2 h, 1 day	Basic knowledge test	For all indicators: Yes
							Applied knowledge test (triage accuracy)	
							Observed performance of managing the jaw trust airway maneuver (overall performance score via expert evaluations of success vs. failure)	
Ripoll-Gallardo, 2020 [40]	Single group, pre-post	8	Physicians	General disaster management	Discussion-based training, real-life scenario, educational videos, computer-based learning, field visits	NA, 6 months	Basic knowledge test	Yes
							Observed performance in real-life simulation in a low-resource emergency room (overall performance score assessed with the TIGR [90])	Yes
							Self-rep. attitude towards disaster management domains	No
Rivkind, 2015 [91]	Single group, pre-post	309^3^	Medical students	Trauma management	Lecture, discussion-based training, real-life scenario, practical skills, educational video, computer-based learning, debriefing	77 h, 2 weeks	Basic knowledge test	Yes
Saiboon, 2021 [92]	Single group, pre-post	168	Medical students	General disaster management	Educational video	0.5 h, 7 days	Basic knowledge test	Yes
Scott, 2010 [51]	Single group, pre-post	61	Medical students	General disaster management	Lecture, real-life scenario	3 h, 1 day	Basic knowledge test	Yes (but only descriptive statistics)
							Self-rep. knowledge	
Sena, 2021 [46]	Single group, pre-post	22	Physicians	General disaster management	Lecture, discussion-based training, debriefing	2 h, 1 day	Basic knowledge test	No
							Self-rep. confidence	Yes
							Perceived importance of disaster medicine training	No
Smith, 2015 [64]	Single group, pre-post	65	Nursing students	Nursing leadership skills in disaster response	Lecture, discussion-based training, real-life scenario, VR on screen with movement tracking via webcam	8 h, 1 day	Self-rep. self-efficacy	Yes
Unver, 2018 [44]	Single group, pre-post	87	Nursing students	General disaster management	Lecture, real-life scenario, debriefing	NA, 8 weeks	Self-rep. disaster preparedness	Yes
Wiese, 2021 [59]	Non-RCT (pre-post), cross-over design	80	Nursing students	General disaster management	IG: computer-based learning CG: practical skills, debriefing	NA, NA	Basic knowledge test	IG better than CG
							Perceptions about learning	No sign. difference between groups
Xia, 2020 [56]	RCT (pre-post)	63	Nursing students	General disaster management	IG: lecture, discussion-based training, real-life scenario, educational video, debriefing CG: No intervention	7 h, NA	Basic knowledge test	Partially confirmed
							Self-rep. attitude (one score combining attitudes towards the training, towards disaster preparedness and family disaster preparation)	No sign. difference between groups
Yanagawa, 2018 [32]	Non-RCT (only post)	63	EMTs	General disaster management	IG: Lecture, real-life scenario, practical skills CG: No intervention	NA, 1 day	Observed performance of whole team during real-life simulation (accuracy + overall performance score assessed with a self-developed instrument)	No
Zhang, 2021 [39]	RCT (pre-post)	120	Nurses	General disaster management	IG: VR on screen, discussion-based training, real-life scenario CG: Lecture, discussion-based training, real-life scenario, practical skills	48 h, 3 months	Basic knowledge test	For all indicators: IG better than CG; CG improved as well but within-group effects not tested for significance
							Observed performance during real-life simulation (overall performance score assessed with the self-developed emergency care capability rating scale)	
							Observed performance in technical skills (overall performance score assessed with a self-developed instrument)	
							Self-rep. disaster preparedness	
Zheng, 2020 [33]	RCT (pre-post)	103	Medical students	Triage	IG: Lecture, discussion-based training CG: Lecture	NA, 3 weeks	Basic knowledge test	IG better than CG
							Observed triage performance (accuracy + time) during real-life simulation	No sign. difference between groups
							Satisfaction with training	IG partially better than CG

Sign. = significant, CG = control group, IG = intervention group, HMD = head-mounted display; Self-rep. = self-reported

^1^Number of participants in analyses

^2^Only referring to relevant subsample as there were separate analyses reported

^3^Number refers to trainees from the years 2010–2012

### Research question 1: overview of training methods

**Fig. 2:**
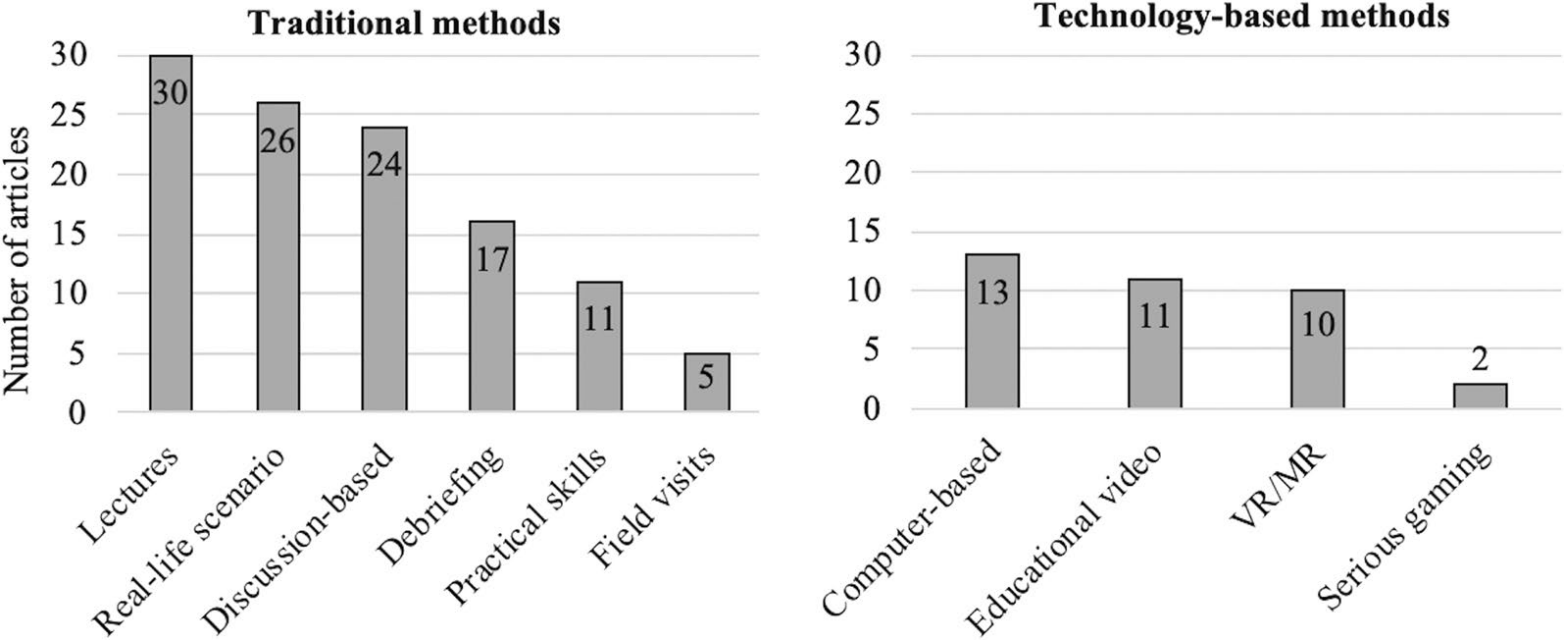
Overview of the distribution of traditional and technology-based training methods

The majority of studies reported trainings that contain a combination of several methods, either in the intervention group, control group, or in both (*k* = 42). Training methods could be categorized into traditional and technology-based methods (Fig. 2). Traditional categories reflect lectures, real-life scenario training (e.g., mass casualty incident simulations with actors or manikins), discussion-based training (including seminars, workshops, in-class games, tabletop exercises), practical skills training (e.g., regional anesthesia), field visits (e.g., the visit of disaster affected sites or riding with the prehospital physician vehicle), and debriefings. In contrast, the technology-based category is composed of computer-based learning (i.e., online learning, educational computer programs), screen-based serious gaming, educational videos, and VR/MR. The term serious gaming refers to computer-based learning that additionally contains game elements, such as cooperation, competition, and stories [24].

### Research question 2: effectiveness indicators

**Fig. 3:**
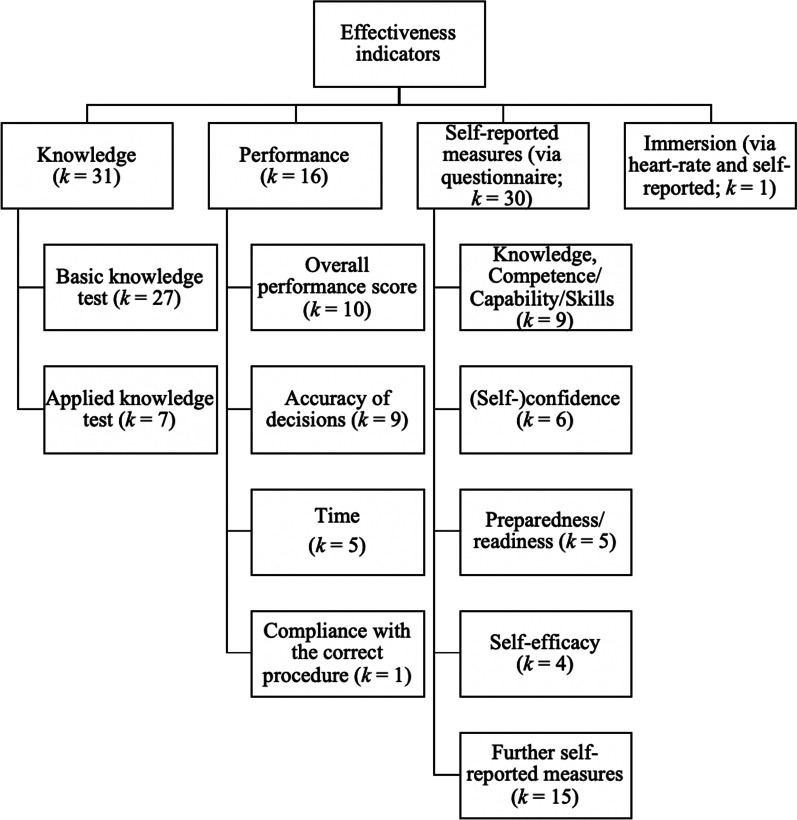
Effectiveness indicators with the number of articles that used them

The trainings were evaluated with several effectiveness indicators, including knowledge and performance, but also self-reported measures (Fig. 3). Most frequently, knowledge gain was used as an indicator. Knowledge was mainly assessed with a basic knowledge test on the training content, often in a multiple-choice format. Some studies used an applied knowledge test that consisted of a written test with several patient descriptions which had to be classified into triage categories. Less than one third of the studies used performance as an indicator. Performance assessments were frequently conducted in triage simulations [25–33] but also in in other contexts, e.g., the management of patients affected by chemical, biological, radiological, nuclear and/or explosive events (CBNRE; 34,35) and the execution of specific medical procedures [36, 37]. Several of those studies focused on measures that could be determined easily and relatively well objectively, including accuracy of triage or treatment decisions [26–33, 38], time needed [28–30, 33, 38] or compliance with the correct procedure [30]. In ten studies, raters composed an overall performance score based on several criteria [25, 28, 32, 34–40], e.g., the evaluation of safety on site [25, 32] and airway/breathing interventions [25, 28]. Three of those studies used already existing assessment instruments, either for treating CBNRE patients [34, 35] or for single patient care in low-resource countries [40]. Only one study used team performance as a measure of effectiveness by letting raters compose an overall team performance score for the management of simulated disaster scenes [32]. All other studies measured individual performance only. Furthermore, several studies used self-reported measures, including preparedness/readiness [39, 41–44] and (self-)confidence [45–50]. In addition to knowledge, performance and self-reported measures, one study compared the level of immersion in VR to real-life scenario training [31].

### Research question 3: effectiveness of training methods

All training methods demonstrated a certain effectiveness, as most studies reported positive or at least partially positive effects of the different methods (see Table 2 for an overview of the methods’ effectiveness).

**Table 2:** Effectiveness of methods

Method	Indicator	Confirmed	Partially confirmed	No effect found
Lectures	Knowledge	**57**, 36, 41, 51, 63, 89, 91	53, 56, 82	52, 46, 62, 85
	Performance	**28,** 26, 29, 36		32
	Self-rep. preparedness	41, 43, 44		
	Self-rep. knowledge and competence	71, 51, 52, 54		
	Self-rep. confidence	46		
	Self-rep. self-efficacy	64		
	Further self-rep. measures	43, 54, 55		53, 46, 56
Real-life scenario training	Knowledge	34, 63, 40, 51, 91, 89	53, 56, 82	45
	Performance	34, 26, 27, 40, 29		32
	Self-rep. preparedness	43, 44		
	Self-rep. knowledge and competence	45, 51		
	Self-rep. confidence	45, 47		
	Self-rep. self-efficacy	34, 64		
	Further self-rep. measures	34, 43, 47, 84,55		40, 56, 53
Discussion-based learning	Knowledge	79, 34, 83, 41, 36, 63, 40, 91, 33	53, 56, 82	52, 46, 85
	Performance	34, 36, 40		
	Self-rep. preparedness	41		
	Self-rep. knowledge and competence	**86, 88,** 71, 52	49	
	Self-rep. confidence	46	49	
	Self-rep. self-efficacy	34, 64		
	Further self-rep. measures	34, 55		53, 40, 46, 56
Practical skills training	Knowledge	79, 37, 91, 41		85
	Performance			32
	Self-rep. preparedness	41		
	Self-rep. knowledge and competence	54, 71		
	Self-rep. confidence			
	Self-rep. self-efficacy			
	Further self-rep. measures	54		
Field visit	Knowledge	63, 40		
	Performance	40		
	Self-rep. preparedness			
	Self-rep. knowledge and competence	54, 71		
	Self-rep. confidence			
	Self-rep. self-efficacy			
	Further self-rep. measures	54		40
Debriefing	Knowledge	26, 27, 63, 83, 91	56	45, 46, 62
	Performance			
	Self-rep. preparedness	44		
	Self-rep. knowledge and competence	45		
	Self-rep. confidence	45, 46, 47		
	Self-rep. self-efficacy			
	Further self-rep. measures	47, 55, 84	**60**	56
Computer-based learning	Knowledge	34, 37, 40, 83, 89, 91	**81,** 82	
	Performance	27, 34, 40		
	Self-rep. preparedness	**42**		
	Self-rep. knowledge and competence		49	
	Self-rep. confidence		49	
	Self-rep. self-efficacy	**42, 81,** 34		
	Further self-rep. measures	34		40
Educational videos	Knowledge	**35, 92,** 41, 63, 40, 37, 91	56, 82	52
	Performance	40	**35**	
	Self-rep. preparedness	41		
	Self-rep. knowledge and competence	52		
	Self-rep. confidence			
	Self-rep. self-efficacy			
	Further self-rep. measures			40, 56
VR (on screen)	Knowledge	63		62
	Performance	29		
	Self-rep. preparedness			
	Self-rep. knowledge and competence			
	Self-rep. confidence			
	Self-rep. self-efficacy	64		
	Further self-rep. measures			

Bold numbers indicate that the study tested the method as a sole training method; Self-rep. = self-reported; *Self-reported preparedness* also covers readiness; *Self-reported knowledge and competence* also covers self-reported capability and skills; Note that the table only lists studies that test pre-post comparisons of a training, while between-method comparisons are described in the text; The categories *serious gaming* and *immersive VR* are not part of this table because the studies in which they were used focused on between-method comparisons

*Lectures* were mostly used in combination with other methods and often served the initial theoretical knowledge transfer [46, 51–56]. There were three studies in which only lectures occurred between the pre-test and post-test. Two of these evaluated educational refresher sessions and reported a positive impact on knowledge [57] and performance [28]. The third one concluded that lectures led to similar performance but lower knowledge gain and partially lower training satisfaction than the combination of lectures and discussion-based training [33]. Multimethod trainings with lectures showed mixed results regarding knowledge and performance but positive effects on self-reports of preparedness, knowledge, competence, confidence, and self-efficacy.

*Real-life scenario training* was often similarly or less effective compared to technology-based training. Studies that compared real-life scenario training to either educational videos [58] or VR [31] reported a partially lower impact of real-life practice on knowledge [58] and similar impacts on performance [31] and training satisfaction [31]. In combination with other methods, the training also resulted in similar [29] or slightly lower [25] performance but greater knowledge gain [25] than VR training and lower self-reported competence than serious gaming [24].

*Discussion-based learning* was often combined with other methods and resulted in mixed knowledge outcomes but at least partially positive effects on performance and self-reports of preparedness, competence, confidence, and self-efficacy. However, two studies reported smaller performance improvements [30] and self-reported competence gain [24] than trainings that contained serious gaming.

*Practical skills training* was never tested as a sole method. Compared to technology-based training, multimethod training with practical skills exercises always resulted in similar or smaller effects. Trainings containing practical skills exercises led to similar [37] or lower [59] knowledge gain as well as similar performance levels [37] and self-reported learning gains [59] than trainings that contained computer-based learning instead. Furthermore, multimethod training with practical skills exercises resulted in lower performance, self-reported preparedness, and self-reported competence than screen-based VR [39] and lower self-reported competence than serious gaming [24].

*Field visits* were part of five trainings and varied considerably in their content and length. Evidence suggests positive effects on knowledge, performance, and self-reports of knowledge and competence. One paper compared a visit of a large ambulance bus to VR and MR training and concluded that the visit was less effective in increasing performance [38]. However, trainees only had one hour in the ambulance bus to practice finding essential objects while the VR and MR group could practice as many times as they wanted within one week (at least three times).

*Debriefings* were only explicitly tested once. The study used drone videos from a real-life scenario training that the trainees had previously undergone [60] and partially confirmed a positive effect on (self-)perception. In combination with other methods, debriefings led to positive outcomes on performance as well as on self-reports of knowledge, confidence, and preparedness. There were mixed findings regarding objectively measured knowledge. Furthermore, multimethod training with debriefings led to lower knowledge scores and similar self-reported learning gains than computer-based learning [59] as well as lower self-reported competence than serious gaming [24].

*Computer-based learning* as a stand-alone method or in combination with other methods led to improvement or partial improvement in knowledge, performance, and self-reports of preparedness, competence, and self-efficacy. Computer-based training resulted in greater knowledge gain and similar self-reported learning gains compared to traditional training [59]. Computer-based learning also led to similar knowledge and performance improvements as practical skills training, both combined with videos [37].

*Educational videos* usually led to at least partial knowledge gain and performance improvements as well as a partially greater knowledge gain than real-life scenario training [58]. Only one multimethod study did not find an effect on knowledge. Studies also reported positive outcomes on self-reported preparedness and competence.

*Serious gaming* was only evaluated in two studies [24, 30]. Ma and colleagues reported that game-based teaching resulted in significantly higher self-reported disaster nursing competence than traditional training [24]. Knight and colleagues tested a multimethod training including a lecture and serious gaming within VR [30]. Compared to traditional training, it fostered better triage accuracy and partially better step accuracy. The time needed to triage did not differ between groups.

### Research questions 3 and 4: current role and effectiveness of VR and MR

**Fig. 4:**
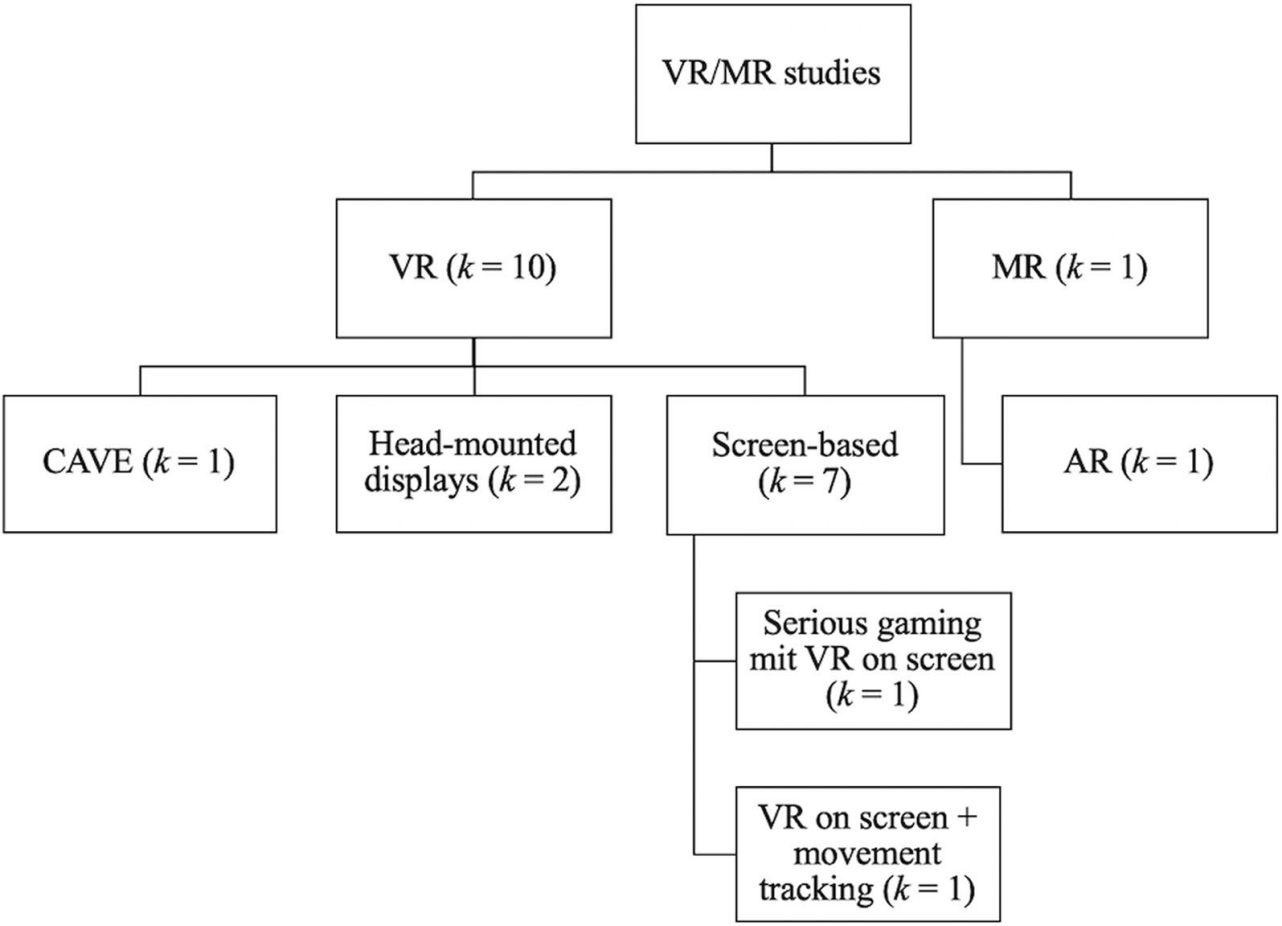
Overview of VR/MR studies

The VR/MR training systems were mostly used for MFR groups with little or no work experience, including students [29, 31, 61–64], cadets [38] or job starters [25]. Seven studies tested trainings that contained PC-screen-based VR (Fig. 4), although always in combination with other methods [29, 30, 39, 61–64]. Five of them covered the topic of triage [29, 30, 61–63], two decontamination [61, 64], one the management of COVID-19 patients [39], and one general disaster scene management [63]. The virtual scenarios mainly included manmade disasters such as traffic accidents [29], explosions in busy areas [30, 61], building collapse and fire on boats at a seaport [63] while one simulated a major earthquake [62]. Two studies that tested pre- and in-hospital trainings used either scenarios in both settings [39] or only an in-hospital scenario [64].

During the VR exercises, trainees were able to move their avatar around and perform a variety of intervention, e.g., breathing/airway checks [29, 30, 62]. While the participants usually used a mouse, keyboard and/or a joystick, one screen-based VR system tracked the trainees’ movements with a webcam as they performed decontamination exercises [64]. Training that contained screen-based VR led to mixed findings regarding knowledge but to positive performance and self-efficacy outcomes. Compared to exclusively traditional trainings, training with screen-based VR led to greater knowledge gain [39] and self-reported preparedness [39] as well as partially greater [30, 39] or similar performance levels [29]. Furthermore, the combination with computer-based learning led to greater knowledge gain than computer-based learning alone [61].

Three studies evaluated immersive VR technology [25, 31, 38]. The first one evaluated triage training in a VR CAVE [25]. The scenario was an explosion in an office building. To perform the triage, trainees observed virtual patients to assess their respiratory rate and verbally requested pulse rates. In terms of training effectiveness, the VR exercise resulted in slightly better performance but poorer knowledge scores than real-life scenario training, both in combination with lectures. Two studies evaluated VR training with head-mounted displays in which trainees used controllers to interact with their virtual surroundings [31, 38]. The first one tested a triage training with a car chase and shooting scenario [31]. Participants could click on icons attached to each casualty to gather basic clinical information and allocate triage cards. The other VR training was designed to help MFRs get a better orientation in a large ambulance bus by practicing to find essential medical equipment [38]. Both VR systems provided feedback regarding the correctness and time of task execution. Overall, these two VR trainings with head-mounted displays led to similar [31] or greater [38] performance than traditional training and to a similar learning satisfaction [31]. One of those studies, however, indicated a higher immersion level during real-life simulations which seemed to be caused by the subscale physical demand [31]. The only study using MR compared AR training to VR with head-mounted displays and traditional education [38]. The AR training was completely similar to the VR ambulance bus training except for the use of an AR headset with transparent lenses. The device projected holograms in the trainees’ field of view. With click gestures, they were able to interact with their environment, like opening/closing drawers. The AR training resulted in a better performance than traditional training, but not as much as the VR training.

### Quality assessment

Overall, the study quality was satisfactory (for a detailed overview see Additional file 1: Tables 2 and 3). For the experimental studies, either none (*k* = 9) or one question (*k* = 5) out of 13 were answered with *no*. For the quasi-experimental studies, usually none (*k* = 4), one question (*k* = 23) or two questions (*k* = 13) were answered with *no*. There was only one paper for which four out of nine questions were answered in the negative [46]. The higher risk of bias in the quasi-experimental studies was mainly based on question 4, which assesses the control group because a large part of the studies had a single group pre-post design (*k* = 35). Furthermore, some studies did not have a complete follow-up or a detailed explanation or analysis for the dropout (*k* = 9).

## Discussion

Well-trained MFRs are essential for managing disaster situations with multiple casualties [3, 4]. To ensure that future disaster training is as effective as possible, we conducted this review on scientifically-evaluated trainings which comprised both traditional and technology-based methods. The trainings were evaluated with several different effectiveness indicators, including knowledge, performance, self-reported measures, and immersion. Despite the heterogeneity of methods and outcome measures, some conclusions could be synergized. While all methods demonstrated effectiveness, the results of this review suggest that technology-based methods often lead to similar or greater training outcomes than exclusively traditional training. Furthermore, we found ten studies that used VR, although usually combined with other methods and often PC-screen-based. Only one study evaluated MR training [38].

Although trends in effectiveness could be identified, the data basis was not sufficient to declare some methods as unequivocally more effective than others. Training methods were often tested in combination, which impaired drawing unbiased conclusions about individual methods. Furthermore, the various effectiveness indicators that were used had only limited comparability. Fewer than one-third of the included studies used performance observation as an evaluation tool. Instead, several studies used knowledge tests or self-assessments (e.g., confidence) although these have limited predictive value for actual performance [65–67]. Despite the great variety in studies, the data basis strongly suggests the strength of technology-based methods. Several studies compared technology-based training to training with real-life scenario exercises which are usually considered the gold standard of disaster training [68]. While these studies suggest the great potential of technology-based methods, there may be a certain degree of bias. Real-life scenario training often served as (part of) the exclusively traditional training for control groups. Therefore, studies may not have been published that did not find at least an equivalent effect of their newly developed technological methods. Instead, the training technology might have been improved and retested until it was similarly or more effective, leading to a publication bias. The same might apply to practical skills training which was always used in combination with other methods and resulted in similar or lower training effectiveness than trainings that contained technology-based methods.

Generally, the current literature indicates that technology-based methods are well suited to train MFRs for disasters. Given the usually limited resources of MFR organizations, these methods promise to be particularly beneficial. Although initial investment in the technology is required, it can then be used flexibly and repeatedly. Thus, a higher, more individually adapted training frequency can be created than with many traditional methods, especially real-life scenario training.

### Current use of VR/MR and its future potential

Seven out of ten studies that tested VR training focused on non-immersive, screen-based VR. The advantage of screen-based VR is that usually no hardware other than normal computer accessories is required. However, more immersive trainings offer greater similarity to experiencing real disaster situations and could therefore be even more useful for preparing MFRs for stressful and unfamiliar situations. Given that high stress can affect the performance of MFRs, training should explicitly address stress responses [69, 70]. Although some of the reviewed trainings contained in-class teaching about dealing with emotions or stress (e.g., [39, 55, 71]), we found no studies that explicitly conducted scenario-based training while assessing and controlling for stress responses. To provide more insight into behavioral changes under stress, future studies should conduct and evaluate explicit disaster training with (continuous) stress measurements to investigate its potential for MFRs. The ongoing improvement of immersive VR and MR technology [72] seems quite promising as it can provide increasingly realistic immersive training scenarios with fewer organizational demands than real-life simulations regarding time and space. Users can experience and practice an almost unlimited number of scenarios in which demands and difficulty levels can be designed as needed [73]. Our results indicate that practical exercises with immersive technology can be conducted nearly everywhere, at any time, and with relatively little preparation, i.e., without setting up a real disaster scene. Furthermore, technical progress in recent years now allows several people to interact within the same virtual environment [74] and treat patients together as in realistic rescue operations.

### Future research

Given the heterogeneity of the current literature, future research should further investigate the effectiveness of individual training methods but also systematically assess whether certain combinations work particularly well. Furthermore, training methods and validated training evaluation tools should be developed not only in terms of effectiveness, but also in terms of efficiency as (financial) resources are often limited. The results of this review suggest, for example, that technological methods such as serious gaming and VR are similarly good or better than traditional methods so that complex real-life scenario trainings with actors could be at least partially replaced. There is also initial evidence that lectures, as an easily implemented method, are well suited for refresher sessions. Future research still needs to clarify the usefulness of immersive VR and especially MR as we only found one MR experimental study that matched our inclusion criteria.

The effectiveness-efficiency trade-off also applies to training evaluation. While knowledge tests offer the advantage of being very easy to conduct and evaluate, the transferability of training success to actual operations is unclear. Performance evaluations during (virtual or real-life) scenario training may be more suitable as they are closer to the target behavior of MFRs during disasters. This review has already identified some indicators, including accuracy of decisions, time needed and compliance with the correct procedure. Future research should focus on finding the appropriate performance measures for diverse disaster training contents in terms of resource efficiency, usability, and relevance. New training technologies could also provide further opportunities for performance assessment, e.g., eye-tracking to gain insights into attentional processes. Furthermore, the assessment of team performance has hardly been considered in disaster training research, although MFRs mainly work in teams. Disaster management is a team effort and is often done in ad-hoc teams similar to other domains of acute care medicine [75]. Improved and trained teamwork improves medical performance [76]. Future studies should also assess long-term benefits of the different training methods and their combination as most of the studies we found only conducted pre-post testing within a few days or weeks.

### Limitations

Our review has three main limitations. First, we only included studies published in English so we might have missed relevant studies published in other languages. Second, we only kept studies in which it was either evident that the sample only consisted of MFRs or in which separate analyses for MFRs were provided. This led to the exclusion of some studies with insufficiently specified sample categories such as *others*. However, it might be possible that the participants were also MFRs. Third, we decided to include only quasi-experimental and experimental studies. We consider this a strength of this systematic review, as it allowed us to create a better overview of the trainings’ effectiveness. Nevertheless, we cannot draw conclusions about what training methods are generally used in disaster training research and whether new methods have been added without being tested in (quasi-)experiments.

## Conclusion

We found several traditional and technology-based trainings methods. The trainings were mainly evaluated with knowledge tests and self-reported measures, while less than one third also used actual performance measures. For valid and yet inexpensive evaluations, objectively assessible performance measures, such as accuracy, time, and order of certain actions can be used. In this review, we found that technology-based methods were often similarly or more effective than traditional training. They therefore offer great potential to supplement or at least partially replace traditional training as especially the organization of the gold-standard, real-life scenario training, can be costly and time-consuming. Two training technologies that have become increasingly popular and affordable are VR and MR. This review suggests that they have great potential which is why further assessments of these technologies are required.

## Supplementary Information

**Additional file 1:** Search string, additional study information and risk of bias.

## Data Availability

All data generated or analyzed during this study are included in this published article and its supplementary information files.

## Abbreviations

AR: Augmented reality
CBNRE: Chemical, biological, radiological, nuclear and/or explosive events
JBI: Joanna Briggs Institute
MFR: Medical first responder
MR: Mixed reality
RCT: Randomized controlled trial
VR: Virtual reality
