# Supplementary material for: Preparing medical first responders for crises: a systematic literature review of disaster training programs and their effectiveness
Source: Scand J Trauma Resusc Emerg Med. 2022 Dec 24;30:76. doi: 10.1186/s13049-022-01056-8 (PMC9789518; doi:10.1186/s13049-022-01056-8)
Supplement: Supplementary file 1 — Additional file 1: Search string, additional study information and risk of bias. [file 13049_2022_1056_MOESM1_ESM.pdf]

Additional File

Search String

Pubmed

(("Health Personnel"[MeSH Terms] OR "Emergency Medical Technicians"[MeSH Terms] OR "Emergency Medical Services"[MeSH Terms] OR "Emergency Medicine"[MeSH Terms] OR "first responder*"[Title/Abstract] OR "emergency medical technician*"[Title/Abstract] OR "ambulance"[Title/Abstract] OR "paramedic*"[Title/Abstract] OR "prehospital"[Title/Abstract] OR "nurse*"[Title/Abstract] OR "nursing student*"[Title/Abstract] OR "medical student*"[Title/Abstract] OR "physician*"[Title/Abstract] OR "health care"[Title/Abstract] OR "healthcare"[Title/Abstract] OR "emergency service*"[Title/Abstract])

AND ("Teaching"[MeSH Terms] OR "Education"[MeSH Terms] OR "Simulation Training"[MeSH Terms] OR "training*"[Title/Abstract] OR "practice*"[Title/Abstract] OR "exercise*"[Title/Abstract] OR "education*"[Title/Abstract] OR "teaching*"[Title/Abstract] OR "simulation*"[Title/Abstract])

AND ("Disasters"[MeSH Terms] OR "disaster medicine/education"[MeSH Terms] OR "Mass Casualty Incidents"[MeSH Terms] OR "mass casualt*"[Title/Abstract] OR "disaster*"[Title/Abstract] OR "major incident*"[Title/Abstract] OR "major accident*"[Title/Abstract] OR "catastrophe*"[Title/Abstract])

AND ("Comparative Study" [Publication Type] OR "Evaluation Study"[Publication Type] OR "Outcome Assessment, Health Care"[MeSH Terms] OR "intervention*"[Title/Abstract] OR "effective*"[Title/Abstract] OR "compar*"[Title/Abstract] OR "evaluat*"[Title/Abstract] OR "measure*"[Title/Abstract] OR "assess*"[Title/Abstract])

AND (2010/1:2021/9[pdat]) AND (english[Filter]))

Web of Science

TS=((health personnel OR emergency medical service* OR emergency service* OR emergency medicine OR first responder* OR emergency medical technician* OR ambulance OR paramedic* OR prehospital OR nurse* OR nursing student* OR medical student* OR physician* OR health care OR healthcare)

AND (training* OR practice* OR exercise* OR education* OR teaching* OR simulation*)

AND (mass casualt* OR disaster* OR major incident* OR major accident* OR catastrophe*)

AND (compar* OR evaluat* OR intervention* OR effective* OR measure* OR assess*))

Language: English; Time Span: 2010-01-01 – 2021-09-30

Supplementary Table 1. Table with additional information about studies.

First author, year	Pre- or pre- and in-hospital	Location	Mean age (SD)	Age range	Gender (% female)	Training Scenarios	Results (long)
Aghababaeian, 2013 (58)	Prehospital	Iran	31.32 (3.9)	*NA*	*NA*	Reconstructed accident	There was no significant difference in knowledge increase between groups directly or 15 days after the training. Groups did not significantly differ in their mean performance increase directly after their trainings but the IG performed slightly better than the CG after 15 days (*p* =.02)
Alenyo, 2018 (77)	Prehospital	South Africa	33.14 (7.77)	*NA*	49.6	NA	After the training, the overall correct triage score (pre: 53.9%, post: 63.6%) and overtriage rate (pre: 31.4%, post: 17.9%) improved significantly, while undertriage rates increased (pre: 13.8%, post: 17.8%; for all three no overlap in confidence intervals: *p* <.05).
Alim, 2015 (78)	Both	Indonesia	20.66 *(NA)*	18-29	87.70	Earthquake	The mean test scores for undergraduate students (pre: 9.84, post: 14.46) and diploma students (pre: 10.83, post: 14.68) improved significantly (max. range: 0-20; both *p* = 0.001).
Aluisio, 2016 (79)	Prehospital	USA	*NA*	*NA*	83.3	NA	The mean knowledge score improved significantly (pre: 79.2%, post: 88.4%, *p* < 0.001).
Andreatta, 2010 (25)	Both	USA	*NA*	*NA*	*NA*	Explosion in office building	After training, the CG had a greater knowledge improvement than the IG (Cohen's *d* = 0.63). The IG demonstrated a better triage performance (Cohen's *d* = 0.25; effect sizes only).
Andreatta, 2015 (34)	Prehospital	USA	*NA*	*NA*	*NA*	Exposure to nerve agents	There were significant increases in scores for knowledge, performance, self-efficacy, and affect after training for both groups (all *p* < 0.001). There were no significant differences in any of the post-training outcomes for the two groups.
Bajow, 2016 (63)	Both	Italy	23.6 (1.9)	*NA*	48.28	Building collapse, fire on boats at a seaport	The mean knowledge score improved significantly (pre: 41.0 %, post: 67.7 %, *p* < 0.0001).
Betka, 2021 (49)	Both	USA	*NA*	*NA*	*NA*	Agriculture disaster simulation	*Separate analyses for relevant subsamples;* After training, nursing students reported significantly increased interprofessional collaborative competencies (*t*(9)=7.673, p<.001), disaster management competencies (*t*(9)=8.938, p<.001), and self-confidence (*t*(8)=5.893, p<.001). There was no significant improvement of medical students' interprofessional collaborative competencies or self-confidence but they reported increased disaster management competencies (*t*(6)=6.075, *p* =.001)
Chan, 2010 (71)	Prehospital	China	*NA*	NA	88.2	NA	The Wilcoxon signed rank test revealed that the mean competency score was significantly improved after training (*Z*=-9.02, *p* < .001, Cohen's *d* = 2.79).

Chandra, 2014 (52)	Both	USA	*NA*	18 - 64	71	NA	From pre- to post-training, self-reported capability increased from 71% to 90% (p < .01). There was no significant increase of knowledge.
Chou, 2021 (53)	Prehospital	Taiwan	*NA*	*NA*	54.17	Earthquake	Four knowledge domains were tested with possible scores between 0 and 2. The mean knowledge score increased significantly in the safety domain (pre: 1.22, post: 1.91, p < .0001) and decreased in disaster patient care (pre: 1.83, post: 1.44, p = .0005). There were no significant differences in the mean scores for the communication or resource management domains. There was no significant improvement of willingness to pursue further training or interest in disaster training exercises.
Cicero, 2012 (26)	Prehospital	USA	*NA*	*NA*	*NA*	School shooting, playground violence, school bus crash	Mean triage performance improved from 6.9 before training to 8.0 out of 10 patients accurately triaged one week after the training (p < .0001). Five months later, there was maintenance of triage improvement, with a mean triage score of 7.8 (n = 42; p < .0001).
Cicero, 2017 (27)	Prehospital	USA	*NA*	*NA*	*NA*	Multi-family house fire, school shooting, school bus crash	The median score of triage accuracy improved significantly between baseline and the posttest two weeks after the training (pre: 80%, post: 90%, p < 0.001). There was no significant difference between the posttest and follow-up 6 months later.
Cowling, 2021 (45)	Prehospital	South Africa	*NA*	*NA*	*NA*	Structural collapse	There was no significant improvement in knowledge test scores after training. After the training, participants reported increased self-reported knowledge (p < 0.001) and confidence (p < 0.001).
Cuttance, 2017 (57)	Prehospital	Australia	*NA*	*NA*	46.92	Traffic accident of a minibus	Compared to the CG, all other groups had a significantly greater number of correctly triaged cases (p < .001) and lower under-triaging rates (p < .001). The CG had an accuracy rate of 47%. The provision of either an educational refresher lecture or aide-memoir significantly increased the accuracy rate to 77% and 90%, respectively. Participants who received both the lecture and aide-memoir had an overall accuracy rate of 89% which is significantly higher than the rate of those only receiving the lecture (p = .02). Over-triage rates were found not to differ significantly across any of the groups.
Dittmar, 2018 (28)	Prehospital	Germany	*NA*	*NA*	*NA*	*NA*	*Separate analyses for subgroup;* One year after the first training and before the re-training, paramedics' overall performance score was 90%. After the re-training, it improved to 97% (p < .05). While the overall performance components accuracy, sensitivity, (critical) under-triage, airway and bleeding management significantly improved, the components specificity, (critical) over-triage, and time requirement did not.

Edinger, 2019 (81)	Prehospital	USA	*NA*	*NA*	*NA*	*NA*	Mean knowledge improved significantly (overall pre: 66%, post: 81%, for 4/14 items $p < .05$). Self-efficacy also improved (for all 10 items $p < .05$).
Farra, 2013 (61)	Prehospital	USA	*NA*	18 - 57	91	Radioactive and explosive events	Overall, the main effect of being in the IG on knowledge was significant ($p < .0001$). Although the two groups already differed before training (IG: 13.5, CG: 11.3, max. Range: 0-20, p = .023; Cohen's $d = .964$), the use of Generalized Estimating Equations controlled for these differences. Both groups showed a similar improvement following the training (posttest; IG: 17.68, CG: 16.24). After two months, the IG's knowledge scores demonstrated stability over time while the CG showed significant decay (follow-up; IG: 16.95, CG: 14.10).
Fernandez-Pacheco, 2017 (60)	Prehospital	Spain	29 (5)	*NA*	57	*NA*	After watching the video of the drill, they participated in, 80% of the students modified their self-perception ($p = .001$). The number of behaviors and moments that the students were able to describe increased (behaviors: increase of 14%, $p = .031$; moments: increase of 40%, $p = .033$). Scores in the other variables (thoughts, feelings, strengths, and weaknesses) did not change significantly.
Foronda, 2016 (62)	Prehospital	USA	*NA*	18 - 39	100	Earthquake	There was no statistically significant difference between the group's pre- and post-test performance ($p = .168$).
Furseth, 2016 (50)	Both	USA	nursing students: 21 *(NA)*, para-medic students: 26 *(NA)*	19 - 45	67.2	Large outbreak of food poisoning aboard a cruise ship, an explosion/bombing, a bus crash	Nursing students in the IG had a greater change in attitudes (both $p = .001$), self-confidence ($p < .001$) and satisfaction ($p < .001$) than in the CG. Regarding paramedic students' attitudes, self-confidence and satisfaction, there was no significant difference between the two trainings.
Greco, 2019 (47)	Prehospital	USA	*NA*	*NA*	*NA*	Toxic chemical spill caused by a train derailment	After training, perceived importance of ethical reasoning ($t(89)=-2.832$, $p = .006$) and confidence ($t(89)=−6.609$, $p < 0.001$) increased significantly.
Huh, 2019 (41)	Both	South Korea	23.12 (5.19)	*NA*	83.3	Earthquake, explosion at a marathon tournament (both part of in-class learning)	After training, the IG had a greater improvement in disaster nursing knowledge ($t(58) = 14.37$, $p < 0.001$), triage knowledge ($t(58) = 7.90$, $p = 0.002$) and disaster readiness ($t(58) = 10.82$, $p < 0.001$) than the CG.
Hutchinson, 2011 (82)	Both	USA	*NA*	*NA*	*NA*	Explosion in a chemistry lab (after posttest)	Mean knowledge scores for two out of three groups of nursing students were significantly higher after training than before. The sophomore students' mean knowledge increased from 60.97 to 95.60 ($n = 26$; $p < .05$) and the senior nursing students' from 49.97 to 85.70 ($n = 24$; $p < .05$). Junior-level students' knowledge did not increase significantly (from 54.81 to 93.52; $n = 31$).

Ingrassia, 2015 (29)	Prehospital	Italy	*NA*	*NA*	*NA*	Car accident	On day 1, the group A live scenario triage accuracy was 58% and the average time to assess all patients was 4'28 min per participant. For group B, the overall virtual scenario triage accuracy was 52% and the average time to complete the assessment was 5'18. There was no statistical difference between the two groups. On day 3, the overall triage accuracy for group A in the virtual simulation was 92% and the average time was 3'53. In live exercise, group B performed the triage with an overall accuracy of 84% in 3'25. Again, there was no statistical difference between the two groups. However, there was an equivalent significant improvement between the pre- and the postintervention triage scores (day 1 vs. Day 3, $p < 0.001$). The time to complete each scenario decreased from day 1 to day 3 in both groups ($p < 0.05$). There was a significant improvement between the day 1 and day 3 life-saving treatment scores in both groups (day 1 vs. Day 3, $p < 0.01$).
Ingrassia, 2014 (83)	Both	Italy	*NA*	*NA*	61.5	Car accident	After training, the mean knowledge score increased from 3.95 to 8.29 out of 10 ($p < .01$). Triage accuracy improved from 45% to 78% ($p < .01$).
James, 2021 (84)	Prehospital	USA	25.06 (7.74)	*NA*	94.10	House contamination/ bioterrorism, explosion at a fertilizer plant, camping chaos post-tornado	After training, participants reported more positive attitudes toward teamwork in training ($t(33)=-4.25$, $p < .01$).
Jones, 2014 (43)	Prehospital	USA	*NA*	*NA*	22	Active shooter incident	After training, more participants felt prepared to respond to an active shooter incident (pre: 41%, post: 89%) and comfortable working jointly on rescue operations with law enforcement personnel in response to an active shooter incident (pre: 61%, post: 93%; only descriptive statistics reported).
Kim, 2020 (55)	Both	South Korea	22.82 (1.38)	*NA*	85.3	Gas explosion	After training, participants reported improved response attitudes ($t(33)=16.31$, $p < .001$).
Knight, 2010 (30)	Prehospital	United Kingdom	*NA*	*NA*	28.57	Bomb explosion in a busy urban street;<br><br>domestic outdoor gas explosion accident (scenario in evaluation)	After training, triage accuracy was significantly higher in the IG than in the CG ($Chi^2 = 13.126$, $p = 0.02$). 72% of the IG and 55% of the CG correctly triaged all 8 patients. Groups did not differ in the number of patients that received correctly followed procedure ($p > .05$). However, more trainees of the IG followed correct procedure for all eight patients ($Chi^2 = 5.45$, $p = 0.0196$). There was no significant difference in time to triage all casualties ($p = 0.155$).

Koca, 2020 (42)	Both	Turkey	IG: 21.16 (1.23) CG: 20.81 (2.47)	*NA*	77.45	Earthquake, fires and combination of both	After training, the IG had significantly higher preparedness and self-efficacy scores than the CG. Training explained 33.1% (R^2 = .331) of the increase in disaster preparedness and 31.7% (R^2 = 0.317) of the increase in disaster response self-efficacy.
Koutitas, 2021 (38)	Prehospital	USA	*NA*	*NA*	NA	NA	Compared to the CG, the VR solution helped trainees to increase their skills by a factor of 46% in terms of number of errors, 29% in terms of speed and 36% as an overall performance (a metric that normalizes the error and speed metric in one formula). The AR solution improved their skills by a factor of 34.5% in terms of number of errors, 10% in terms of speed and 17% as an overall performance (only descriptive statistics reported).
Kuhls, 2017 (48)	Prehospital	Thailand	Nurses: 40 (*NA*) physicians: 38 (*NA*)	nurses: 24-63 physicians: 25–61	54.94	NA	Separate analyses for relevant subsamples; After the training, all occupational groups reported a significant confidence increase in each confidence category surveyed (all $p <$ .001). Physicians' and nurses' median changes in the different confidence areas were between 1 and 2 points (scale: 1-5).
Lampi, 2013 (85)	Prehospital	Sweden	*NA*	*NA*	*NA*	Bus crash (scenario in evaluation)	There was no significant difference between pre and post-course test results (p >.05).
Lennquist Montán, 2015 (86)	Both	NA	*NA*	*NA*	*NA*	NA	Separate analyses for subsample; A significant increase between pre- and post-course assessment was registered for all items of the self-reported knowledge and skills questionnaire ($p < 0.001$). The average increase for prehospital staff was 74%.
Ma, 2021 (24)	Prehospital	China	IG: 19.22 (0.76), CG: 19.17 (0.80)	*NA*	83.7	Earthquake disaster as the background of the IG game	After training, the IG rated their competency significantly higher than the CG ($t(102)$=3.114, p =.002).
Merlin, 2010 (54)	Both	USA	*NA*	*NA*	*NA*	*NA*	The mean value of the 4 self-reported knowledge items increased from 3.11 before training to 4.35 after training (scale from 1 to 5; 3 out of 4 items p > .0001). Greater than 35% opinion change was found in several areas, including education differences in prehospital providers, general teaching of prehospital care (both $p < 0.0001$).
Mills, 2020 (31)	Prehospital	Australia	*NA*	*NA*	*NA*	Police car chase and shooting	Triage accuracy did not differ in the two tested training methods. Participants needed more time to triage live patients compared to virtual patients (p<0.001). Average heart rate, heart rate increase, and maximum heart rate were significantly higher during the live

							simulation compared to the VR simulation (all p<0.001). Participants reported a significantly higher immersion level during the live simulations (p<0.001) which seems to be caused by the subscale physical demand (p<001, all other subscales p>.05). There were no significant differences in learning satisfaction.
Motola, 2015 (35)	Prehospital	USA	IG: 37.3 (*NA*) CG: 36.9 (*NA*)	*NA*	IG: 4 CG: 7	Nerve agent, explosives, radiologic event	After training, the IG had a significantly greater improvement in knowledge than the CG (IG: 53.3% to 63.4%, CG: 55.5% to 59.3%, p = 0.001). The IG performed better in the explosives and chemical nerve agent scenarios than the CG (both p < 0.01) but there was no significant difference between the groups in the radiologic scenario (p = 0.51).
Paddock, 2015 (37)	Prehospital	USA	IG_1: 43 (13) CG: 50 (11) IG_2: 43 (9)	*NA*	33.33	*NA*	Within each training group, there was a statistically significant improvement in the mean pre- and post-course knowledge test scores (all p < 0.001, pretest: 14.3%-20.8%, posttest: 53%-54.3%). There were no significant differences between the three training groups' post-course knowledge gains (p > 0.05). After the training the groups did not differ in their mean image acquisition scores or their image interpretation scores.
Phattharapor-njaroen, 2020 (88)	Both	Thailand	*NA*	26–35	69.32	Terror attack along with a bomb explosion, riot, and shooting; Building fire	After training, self-reported knowledge increased in all domains (all p < .01) with the greatest improvement in safety issues (pre: 40%, post: 96%) and the lowest in treatment (pre: 54%, post: 71%).
Pollard, 2015 (89)	Both	USA	*NA*	*NA*	*NA*	Plane crash on a soccer field near the airport, carrying terrorists; operating room explosion; patient trapped in debris field resulted from a tornado	The training led to a significant increase in knowledge. This result was found in the subsample that provided complete data ($t(7)$=-2.35, p = 0.05) as well as in the imputed dataset ($t(40)$=-11.72, p < 0.001).
Pouraghaei, 2017 (36)	Prehospital	Azerbaijan	34.97 (6.42)	23 - 50	*NA*	*NA*	After training, the mean triage knowledge score (pre: 11.47, post: 13.63, max. Range: 0-15, p < .05) and the knowledge score in the performance section increased significantly (pre: 10.73, post: 14.93, max. Range: 0-19, p < .05). The number of participants able to perform the jaw trust airway maneuver increased as well (pre: 21.9%, post: 88.3%, p < 0.001).
Ripoll-Gallardo, 2020 (40)	Both	Italy	*NA*	*NA*	37.5	*NA*	After training, there was a significant improvement in knowledge scores (10.4 points, max. Range: 0-30; p = .001) as well as in performance scores (3 points, scale: 1-7; p = .000001). Attitudes did not change significantly.

Rivkind, 2015 (91)	Both	Israel	*NA*	*NA*	NA	*NA*	After training, the mean knowledge score increased significantly (pre: 54%, post: 68%, *p* < 0.001).
Saiboon, 2021 (92)	Both	Malaysia	*NA*	*NA*	80.4	*NA*	After training, the mean knowledge score increased significantly (pre: 6.99, post: 13.31, max. Range 0-20, *p* < 0.001).
Scott, 2010 (51)	Prehospital	USA	*NA*	*NA*	*NA*	Hazardous materials/chemical spill scenario (overturned truck on a rural two-lane highway with a possible chemical exposure and multiple victims)	In 2008, the average of knowledge scores improved from 39% to 58% (*n* = 30) and in 2009 from 47% to 57% (*n* = 31). Self-reported knowledge increased as well (2008: 3.76 to 7.64 out of 10; 2009: 2.52 to 3.76 out of 5; only descriptive statistics reported).
Sena, 2021 (46)	Both	USA	*NA*	*NA*	*NA*	Explosion at a major sporting event with blast injuries	After training, there was no significant increase in knowledge or perceived importance of disaster medicine training but a significant increase in confidence (pre: 2, post: 4, scale: 1-5; *p* = .011).
Smith, 2015 (64)	Both	USA	*NA*	*NA*	*NA*	Radiological bomb explosion in front of a local courthouse	After training, participants reported increased self-efficacy (*t*(64)=8.45; *p* < .001).
Unver, 2018 (44)	Both	Turkey	21.95 (0.26)	*NA*	100	Earthquake	The Wilcoxon signed rank test revealed that there was a significant increase in preparedness after training (*Z* = -7.572, *p* = .001)
Wiese, 2021 (59)	Prehospital	USA	26.5 (3.49)	16 - 37	70.1	Bus encountering a tornado	After training (but before the cross-over), the IG had a higher mean knowledge score than the CG (IG: 20.55, CG: 15.93, possible range: 0-25; only descriptive statistics reported). Furthermore, participants reported significant learning gains, regardless of whether the training was live or virtual (9 out of 12 items: *p* < .05).
Xia, 2020 (56)	both	China	IG: 21.46 (1.34) CG:34.38 (1.35)	*NA*	84.13<br><br>IG: 83.87 CG: 84.37	Earthquake	After training, the IG displayed greater theoretical knowledge scores in the three tested domains (all *p* < .01) and greater skill-related knowledge in two out of three domains (both *p* < .05) than the CG. One month after the training, the IG had higher scores in theoretical and skill-related knowledge in one of the three domains. The groups did not differ in their attitude at any point.
Yanagawa, 2018 (32)	both	Japan	*NA*	*NA*	NA	Collision between a minibus and a common automobile	Teams that included a chief EMT who attended the training as well as teams that included staff members who attended the training did not perform significantly better than teams without (both total performance scores *p* > .05).
Zhang, 2021 (39)	both	China	*NA*	*NA*	90	Covid-19 patients	After training, the IG demonstrated greater improvement in knowledge (*t*(58)=4.783, *p* < .001), performance (*t*(58)=4.416, *p* < .001), technical skills (*t*(58)=2.708, *p* = .008) and disaster preparedness (*t*(58)=5.295, *p* < .001) than the CG.

Zheng, 2020 (33)	Prehospital	China	IG: 24.55 (1.14) CG: 24.52 (1.0)	*NA*	46.60	Traffic accident	After training, the IG scored significantly better in the knowledge test (difference of 3 points, max. Range:0-50; $p < 0.001$). There were no significant differences in performance between groups. The IG reported a higher satisfaction with the course (5 out of 8 items, $p < .05$) but also perceived it as more work and burden (both, $p < .05$)

Notes. IG = intervention group, CG = control group, NA = not available

Risk of Bias

JBI CRITICAL APPRAISAL CHECKLIST FOR RANDOMIZED CONTROLLED TRIALS

1. Was true randomization used for assignment of participants to treatment groups?
2. Was allocation to treatment groups concealed?
3. Were treatment groups similar at the baseline?
4. Were participants blind to treatment assignment?
5. Were those delivering treatment blind to treatment assignment?
6. Were outcomes assessors blind to treatment assignment?
7. Were treatment groups treated identically other than the intervention of interest?
8. Was follow up complete and if not, were differences between groups in terms of their follow up adequately described and analyzed?
9. Were participants analyzed in the groups to which they were randomized?
10. Were outcomes measured in the same way for treatment groups?
11. Were outcomes measured in a reliable way?
12. Was appropriate statistical analysis used?
13. Was the trial design appropriate, and any deviations from the standard RCT design (individual randomization, parallel groups) accounted for in the conduct and analysis of the trial?

Supplementary Table 2. Risk of bias of the experimental studies.

First author, year	1	2	3	4*	5*	6*	7	8	9	10	11	12	13
Andreatta, 2010	no	yes	yes	not appl.	not appl.	not appl.	yes	yes	yes	yes	yes	yes	yes
Cuttance, 2017	yes	yes	yes	not appl.	not appl.	not appl.	yes	not appl.[1]	yes	yes	yes	yes	yes
Farra, 2013	yes	yes	no	not appl.	not appl.	not appl.	not appl.	yes	yes	yes	yes	yes	yes
Huh, 2019	yes	yes	yes	not appl.	not appl.	not appl.	yes	yes	yes	yes	yes	yes	yes
Ingrassia, 2015	yes	yes	unclear	not appl.	not appl.	not appl.	yes	yes	yes	yes	yes	yes	yes
Koca, 2020	yes	yes	yes	not appl.	not appl.	not appl.	yes	yes	yes	yes	yes	yes	yes
Koutitas, 2021	yes	yes	unclear	not appl.	not appl.	not appl.	yes	not appl.[1]	yes	yes	yes	yes	yes
Ma, 2021	yes	yes	yes	not appl.	not appl.	not appl.	yes	yes	yes	yes	no	yes	yes
Mills, 2020	yes	no	yes	not appl.	not appl.	not appl.	yes	not appl.[1]	yes	yes	yes	yes	yes
Motola, 2015	yes	yes	yes	not appl.	not appl.	not appl.	yes	yes	yes	yes	yes	yes	yes
Paddock, 2015	no	yes	yes	not appl.	not appl.	not appl.	yes	yes	yes	yes	yes	yes	yes
Xia, 2020	yes	yes	yes	not appl.	not appl.	not appl.	yes	yes	yes	yes	yes	yes	yes
Zhang, 2021	yes	yes	yes	not appl.	not appl.	not appl.	yes	yes	yes	yes	yes	yes	yes
Zheng, 2020	yes	yes	yes	not appl.	not appl.	not appl.	yes	yes	yes	yes	yes	yes	yes

Notes. *yes* indicates a lower risk of bias, *no* indicates a higher risk of bias; *not appl.* = not applicable; questions 4-6 refer to blinding. Because blinding is hardly or not at all feasible in training evaluation studies, the items were answered with not applicable; [1]RCT studies with only a post-test.

JBI CRITICAL APPRAISAL CHECKLIST FOR QUASI-EXPERIMENTAL STUDIES

1. Is it clear in the study what is the 'cause' and what is the 'effect' (i.e. there is no confusion about which variable comes first)?
2. Were the participants included in any comparisons similar?
3. Were the participants included in any comparisons receiving similar treatment/care, other than the exposure or intervention of interest?
4. Was there a control group?
5. Were there multiple measurements of the outcome both pre and post the intervention/exposure?
6. Was follow up complete and if not, were differences between groups in terms of their follow up adequately described and analyzed?
7. Were the outcomes of participants included in any comparisons measured in the same way?
8. Were outcomes measured in a reliable way?
9. Was appropriate statistical analysis used?

Supplementary Table 3. Risk of bias of the quasi-experimental studies.

First author, year	1	2	3	4	5	6	7	8	9
Aghababaeian, 2013	yes	yes	yes	yes	yes	yes	yes	yes	yes
Alenyo, 2018	yes	yes	yes	no	yes	yes	yes	yes	yes
Alim, 2015	yes	yes	yes	no	yes	yes	yes	yes	yes
Aluisio, 2016	yes	yes	yes	no	yes	yes	yes	yes	yes
Andreatta, 2015	yes	yes	yes	yes	yes	yes	yes	yes	yes
Bajow, 2016	yes	yes	yes	no	yes	yes	yes	yes	yes
Betka, 2021	yes	yes	yes	no	yes	yes	yes	yes	yes
Chan, 2010	yes	yes	yes	no	yes	yes	yes	yes	yes
Chandra, 2014	yes	yes	yes	no	yes	yes	yes	yes	yes
Chou, 2021	yes	yes	yes	no	yes	no	yes	yes	yes
Cicero, 2012	yes	yes	yes	no	yes	no	yes	yes	yes
Cicero, 2017	yes	yes	yes	no	yes	no	yes	yes	yes
Cowling, 2021	yes	yes	yes	no	yes	yes	yes	yes	yes
Dittmar, 2018	yes	yes	yes	no	yes	no	yes	yes	yes
Edinger, 2019	yes	yes	yes	no	yes	no	yes	yes	yes
Fernandez-Pacheco, 2017	yes	yes	yes	no	yes	yes	yes	yes	yes
Foronda, 2016	yes	yes	yes	no	yes	yes	yes	yes	yes
Furseth, 2016	yes	yes	yes	yes	yes	yes	yes	yes	yes
Greco, 2019	yes	yes	yes	no	yes	yes	yes	yes	yes

Hutchinson, 2011	yes	yes	no	no	yes	yes	yes	yes	yes
Ingrassia, 2014	yes	yes	yes	no	yes	yes	yes	yes	yes
James, 2021	yes	yes	yes	no	yes	yes	yes	yes	yes
Jones, 2014	yes	yes	yes	no	yes	yes	yes	yes	no
Kim, 2020	yes	yes	yes	no	yes	yes	yes	yes	yes
Knight, 2010	yes	yes	yes	yes	no	not appl.[1]	yes	yes	yes
Kuhls, 2017	yes	yes	yes	no	yes	yes	yes	yes	yes
Lampi, 2013	yes	yes	yes	no	yes	yes	yes	yes	yes
Merlin, 2010	yes	yes	yes	no	yes	yes	yes	no[2]	yes
Lennquist Montán, 2015	yes	yes	yes	no	yes	yes	yes	no[2]	yes
Phattharapornjaroen, 2020	yes	yes	yes	no	yes	yes	yes	no[2]	yes
Pollard, 2015	yes	yes	yes	no	yes	yes	yes	yes	yes
Pouraghaei, 2017	yes	yes	yes	no	yes	no	yes	yes	yes
Ripoll-Gallardo, 2020	yes	yes	yes	no	yes	yes	yes	yes	yes
Rivkind, 2015	yes	yes	yes	no	yes	no	yes	yes	yes
Saiboon, 2021	yes	yes	yes	no	yes	no	yes	yes	yes
Scott, 2010	yes	yes	yes	no	yes	yes	yes	yes	yes
Sena, 2021	yes	no	yes	no	yes	no	yes	yes	no
Smith, 2015	yes	yes	yes	no	yes	yes	yes	yes	yes
Unver, 2018	yes	yes	yes	no	yes	yes	yes	yes	yes
Wiese, 2021	yes	yes	yes	yes	yes	yes	yes	yes	yes
Yanagawa, 2018	yes	unclear	yes	yes	no	not appl.[1]	yes	yes	yes

Notes. [1]only a post-test; [2]called the outcome “knowledge”/”skills” but only measured self-rated knowledge/self-rated skills.
